# Approach to Patients with Dysphagia: Clinical Insights

**DOI:** 10.3390/brainsci15050478

**Published:** 2025-04-30

**Authors:** Min-Su Kim

**Affiliations:** Department of Physical and Rehabilitation Medicine, Soonchunhyang University Cheonan Hospital, Cheonan 31151, Republic of Korea; 84730@schmc.ac.kr; Tel.: +82-41-570-2220; Fax: +82-41-570-2221

**Keywords:** dysphagia, esophageal, oropharyngeal, parkinsonism, rehabilitation, stroke, swallowing, video

## Abstract

Dysphagia is a commonly encountered condition in clinical practice, with a rising incidence reported particularly in South Korea. It can be broadly classified into oropharyngeal dysphagia and esophageal dysphagia, and distinguishing between the two is crucial for establishing rehabilitation treatment strategies. Oropharyngeal dysphagia frequently occurs in central nervous system diseases such as stroke, dementia, and Parkinson’s disease and has a significant impact on prognosis. Additionally, because there is a high risk of life-threatening aspiration pneumonia in patients complaining of dysphagia, an accurate diagnosis must be made during the early stages of the condition. Patients with oropharyngeal dysphagia may report difficulty initiating swallowing and may experience coughing, choking, nasopharyngeal reflux, aspiration, and a sensation of leftover food in the pharynx during swallowing. Patients with esophageal dysphagia may report a sensation of food getting stuck in the esophagus for a few seconds after the initiation of swallowing. Esophageal dysphagia should be characterized by analyzing whether the foods causing dysphagia are solid, liquid, or both, as well as by the progression of symptoms, whether they are progressive or intermittent; their severity; and associated symptoms such as weight loss, heartburn, or regurgitation. Video fluoroscopic swallowing study (VFSS), fiberoptic endoscopic evaluation of swallowing (FEES), and esophagogastroduodenoscopy (EGD) are invaluable in determining the causes, severity, and treatment strategies for dysphagia. Since swallowing disorders are significant factors influencing the course and prognosis regardless of the type of disease, clinicians should adopt a systematic approach to such disorders.

## 1. Introduction

Dysphagia is a subjective symptom characterized by difficulty or abnormal sensations during swallowing [1]. It is a relatively common symptom experienced by 3% of the general adult population [2], and it serves as a warning sign that requires immediate identification of the precise cause and initiation of appropriate treatment [3]. Structural or motor functional abnormalities may impair the smooth transit of liquids or solids from the mouth to the stomach [4]. Patients may report that they cannot initiate swallowing or experience a sensation of obstruction when food passes through the esophagus [5].

Although dysphagia is more common in older adults, it is not an inevitable consequence of aging. Several studies reported that aging has some effect on reducing swallowing function. For instance, the swallowing reflex may be delayed in older adults, and decreased elasticity of connective tissues may lead to a reduction in the opening of the pharyngoesophageal sphincter, resulting in insufficient anterior–posterior movement of the larynx [5]. However, research on the correlation between age-related swallowing function decline and the onset of dysphagia is limited. Therefore, it is essential to commence a diagnostic process for the underlying disease [6,7].

Dysphagia can be divided into oropharyngeal dysphagia, which occurs from the beginning of swallowing, and esophageal dysphagia, in which fluid or solid food does not move smoothly from the mouth to the stomach. Odynophagia is a painful feeling when swallowing, whereas globus is defined as a persistent or intermittent foreign body sensation in the throat that is not accompanied by pain [8]. Symptoms occur halfway between the thyroid cartilage and sternal notch and often improve with eating. A globus diagnosis occurs in the absence of structural abnormalities, gastric inlet patches, gastroesophageal reflux disease, or major esophageal motility disorder [9]. In this article, we will describe how to approach patients who complain of dysphagia to accurately diagnose the cause.

## 2. Epidemiology of Dysphagia

Dysphagia has been reported as a relatively common symptom and occurs in 8–22% of adults over 50 years of age [10,11,12,13,14]. The prevalence of dysphagia in Australian nursing homes is estimated to be between 40% and 60% [15]. In a postal questionnaire study of the general population at the Mayo Clinic, the prevalence of dysphagia was 7%, though no associated risk factors were analyzed [16]. In a study published by the Mayo Clinic, 10–33% of older people had had difficulty swallowing more than once a week in the past year [5]. The use of proton pump inhibitors, symptoms such as heartburn and acid reflux, and high somatization scores are associated with the occurrence of dysphagia.

In a survey of nearly 6000 members of the 65-and-older population in China, researchers identified that 39.4% of the respondents had dysphagia [17]. A previous study revealed that a total of 36.9%, 40.5%, 54.9%, and 64.5% of the elderly from the UK, Indonesia, Brazil, and China, respectively, had dysphagia when assessed by an Eating Assessment Tool [18]. In a Korean retrospective cohort study of inpatients and outpatients who visited a medical institution for dysphagia between 1 January 2006, and 31 December 2016, the prevalence of dysphagia increased from 635.4 (0.635%) per 100,000 population in 2006 to 1031.6 (1.032%) per 100,000 population in 2016 [19].

## 3. Causes of Dysphagia

To elucidate the cause of swallowing difficulties, it is important to determine whether the symptoms are caused by problems with the pharynx, upper esophagus, esophageal body, or junction of the stomach and the esophagus. It is also necessary to recognize that many diseases can cause a combination of both oropharyngeal dysphagia and esophageal dysphagia; thus, a thorough medical history and drug history investigation should be performed for each patient (Table 1).

### 3.1. Oropharyngeal Dysphagia

In young adults, oropharyngeal dysphagia predominantly occurs due to muscle disorders and esophageal webs. In elderly individuals, the condition generally arises from central nervous system diseases such as stroke, Parkinson’s disease, and dementia [20,21]. When diagnosing oropharyngeal dysphagia, it is necessary to first assess whether the swallowing difficulties are due to a mechanical obstruction or a neuromuscular disorder. Diseases that can cause mechanical obstructions include Zenker’s diverticulum, cricopharyngeal achalasia, head and neck cancers, and complications following surgery and radiation therapy, as well as cervical osteophytes [22], whereas central nervous system diseases associated with oropharyngeal dysphagia include stroke, Parkinson’s disease, dementia, bulbar palsy, and amyotrophic lateral sclerosis [23].

Stroke patients constitute a significant portion of those undergoing rehabilitation treatment for swallowing difficulties [24]. Within three days of stroke onset, approximately 50% of patients experience oropharyngeal dysphagia, with aspiration occurring in half of these cases, leading to pneumonia in about 30% [25]. Patients with oropharyngeal dysphagia must receive nutrition via a tube, as oral intake can result in life-threatening aspiration pneumonia [26]. The symptoms of aspiration pneumonia tend to be more severe than those caused by viral pneumonia, and the likelihood of progression to sepsis is higher [27]. Furthermore, patients with aspiration pneumonia often face permanent disabilities, and this condition has a higher mortality rate than community-acquired pneumonia [28]. Therefore, it is crucial to determine the presence of swallowing difficulties in stroke patients to prevent complications such as pneumonia. Symptoms associated with oropharyngeal dysphagia occur in approximately 50% of Parkinson’s disease patients, with around 95% exhibiting abnormal findings in video fluoroscopic swallowing studies [29]. Oropharyngeal dysphagia can manifest even in the early stages of dementia and Parkinson’s disease, though it mostly occurs as these diseases progress [30].

### 3.2. Esophageal Dysphagia

Esophageal dysphagia can occur due to a variety of causes. Acute dysphagia usually arises after the ingestion of foreign substances, whereas tumors, such as those related to lung cancer and lymphoma, may mechanically compress the mediastinum, leading to swallowing difficulties [31]. Additionally, conditions like gastroesophageal reflux disease and esophageal webs can narrow the lumen of the esophagus, whereas esophageal neuromuscular disorders such as achalasia may present with swallowing dysfunction [31].

In a retrospective study published by an institution in the United States, the prevalence and trends of the underlying diseases causing dysphagia were analyzed in 1371 patients who underwent esophagogastroscopy for dysphagia from 1999 to 2009 [32]. Gastroesophageal reflux disease and eosinophilic esophagitis were the most common underlying diseases, with prevalences of 27.6% and 7.7%, respectively [32]. Studies conducted in Asia indicate that common causes of esophageal dysphagia include squamous cell carcinoma of the esophagus, achalasia, and surgery-related strictures [33,34]. Gastroesophageal reflux disease (GERD) is generally more prevalent in Western countries than in Asia. While Western countries report prevalence rates of 10–20% or higher, Asia typically exhibits lower rates, often less than 10% [35]. This difference is attributed to various factors including lifestyle, diet, and potentially lower awareness of GERD symptoms in some Asian regions [36]. However, it is believed that the prevalence of GERD is increasing in Asia.

## 4. Differential Diagnosis Between Oropharyngeal and Esophageal Dysphagia

When treating patients who complain of swallowing difficulties, determining whether these difficulties are oropharyngeal or esophageal can be beneficial for the establishment of a treatment plan. Since patients with oropharyngeal swallowing difficulties are at higher risk of aspiration and aspiration pneumonia, it is crucial to make a timely and accurate diagnosis in patients experiencing difficulty swallowing. In a study comparing a control group with 36 elderly patients over the age of 70 hospitalized for pneumonia, oropharyngeal swallowing difficulties increased the risk of community-acquired pneumonia by a factor of 12 [37]. A systematic analysis of observational studies also found that, in stroke patients, the risk of pneumonia increased by 3.17 times (RR 3.17, 95% CI, 2.07–4.87) when swallowing difficulties were present [38].

**Figure 1:**
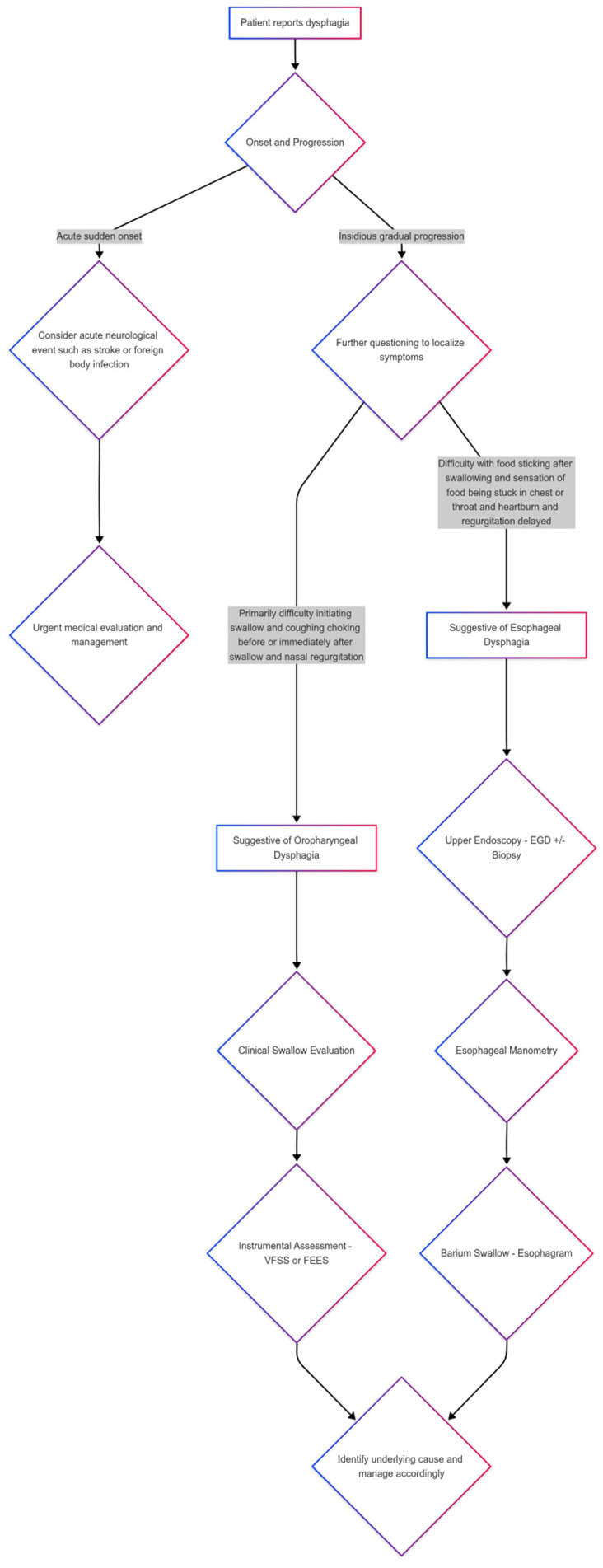
Approach to patients with dysphagia.

Taking detailed histories regarding symptoms significantly aids in differentiating oropharyngeal dysphagia from esophageal dysphagia. Patients with oropharyngeal dysphagia may report difficulty initiating swallowing, recognizing the location of symptoms in the throat, experiencing nasopharyngeal reflux during swallowing, aspiration, or a sensation of food remaining in the pharynx [6]. In contrast, patients with esophageal dysphagia describe difficulty several seconds after initiating swallowing and report a feeling of food being stuck between the upper esophagus and the stomach [31]. When patients indicate the location of the obstruction, it may not correlate with the actual site of the lesion [39]. The approach to patients presenting dysphagia is summarized and illustrated in Figure 1.

## 5. Dysphagia Examination

After taking a detailed history of the patient’s intake, objective tests are conducted to determine the cause of the dysphagia. If oropharyngeal dysphagia is suspected, bedside swallowing tests can be performed to confirm the presence of this condition [40]. To support accurate diagnosis and establish treatment plans, video fluoroscopic swallowing study (VFSS) [21,41], fiberoptic endoscopic swallowing study (FEES) [38], and esophagogastroduodenoscopy (EGD) may be carried out [42].

For patients with swallowing disorders, determining which tests are more appropriate based on age or comorbidities is a significant issue. Many researchers have reported that FEES is a more convenient and cost-effective test for pediatric patients than VFSS [43]. However, depending on the healthcare system of each country and the characteristics of the pediatric patients, VFSS may be preferable. For instance, in the case of newborns who feed using a bottle, swallowing disorders can be easily assessed with VFSS [44]. In Korea, FEES is rarely used for evaluating swallowing disorders due to its high costs. However, it is employed for patients who cannot sit in a wheelchair due to severe joint contracture. Depending on the national health insurance system, FEES can be a more affordable and convenient option for assessing swallowing status. Clinicians can select between VFSS and FEES to assess swallowing disorders in a complementary manner, considering the patient’s characteristics, including age and associated comorbidities, and the health insurance system.

### 5.1. Video Fluoroscopic Swallow Study (VFSS)

The gold standard test for evaluating the swallowing function of patients with dysphagia and directing diagnostic and treatment decisions is the video fluoroscopic swallow study (VFSS) [26,45,46,47]. This test involves the patient consuming food mixed with barium, while real-time X-rays assess the swallowing function. The VFSS demonstrates high reliability and validity in identifying airway aspiration in cases of oropharyngeal dysphagia [48,49]. If there are signs of swallowing disorders, particularly when food enters the airway through the larynx, it is necessary to stop oral intake to prevent pneumonia and insert a Levin tube. Once a swallowing disorder is diagnosed, the patient must fast and rely on tube feeding via a nasogastric tube, which can cause significant distress and may lead to severe depression if prolonged [50]. Prolonged use of silicone nasogastric tubes may irritate the esophageal mucosa, leading to complications such as inflammation or ulcers, making this type of feeding a common cause of life-threatening gastrointestinal bleeding [51].

Prolonged use of silicone nasogastric tubes may irritate the esophageal mucosa, leading to complications such as inflammation or ulcers.

Therefore, if airway aspiration is observed during the VFSS, it is recommended to place an L-tube in the patient and to follow up with VFSS tests every week during hospitalization and every month in outpatient clinics.

### 5.2. Fiberoptic Endoscopic Evaluation of Swallowing (FEES)

Another useful diagnostic test for diagnosing dysphagia is the fiberoptic endoscopic evaluation of swallowing (FEES). The VFSS can only be conducted on patients who can assume a sitting position, requires separate fluoroscopic equipment, and has the disadvantage of exposing the patient to radiation during each examination [21]. FEES involves inserting a flexible endoscope into the nose to directly observe the oropharynx as the patient swallows food, providing several advantages. First, unlike video fluoroscopic examinations that utilize X-ray imaging, it allows for the direct and accurate observation of pharyngeal structures [52]. Second, it enables direct visual assessment of the patient’s swallowing ability and response to excessive pharyngeal secretions [53]. Third, by using the endoscope to directly contact or stimulate the arytenoids, epiglottis, and false vocal folds, one can test whether the laryngeal adductor reflex occurs normally, thereby directly assessing the sensory function of the pharynx [54]. Fourth, during dysphagia rehabilitation, showing the examination directly to the patient through the flexible endoscope can serve as a biofeedback tool for patient education [55]. Fifth, there is no exposure to radiation during each examination, and testing can be conducted regardless of the patient’s position or location [56]. For these reasons, the FEES, in conjunction with VFSS, is a very safe and reliable method for the accurate diagnosis of swallowing disorders. Studies analyzing the prevalence of pneumonia due to aspiration have also reported no diagnostic differences between the two methods, indicating that they should be used complementarily depending on the situation.

### 5.3. Esophagogastroduodenoscopy (EGD)

For patients under the age of 50 who present with typical gastroesophageal reflux symptoms along with dysphagia without warning symptoms, it is recommended to first administer a high dose of proton pump inhibitors for eight weeks [57]. If symptoms do not completely resolve, further examinations should be conducted.

In cases in which esophageal dysphagia is suspected and observed for both solid and liquid food, it is prudent to first consider esophageal motility disorders and perform an esophagography [58]. For patients with dysphagia concerning only solid food, potential conditions such as esophageal rings, eosinophilic esophagitis, gastroesophageal reflux disease, and esophageal cancer should be considered, and an EGD should be evaluated first, with EGD as the initial diagnostic modality [58]. A study in the United States retrospectively analyzed the results of upper gastrointestinal endoscopies in 1649 patients with dysphagia and found that esophageal cancer was diagnosed in 4% of cases, while 54% had identifiable causes for the dysphagia [59]. If the endoscopic findings are normal but there is still a suspicion of mechanical obstruction, such as esophageal rings or external compression, an esophagography should be performed [60]. In patients with suspected esophageal motility disorders or normal endoscopic findings who experience dysphagia, it is essential to perform esophageal manometry [61,62].

## 6. Conclusions

Dysphagia can be classified into oropharyngeal dysphagia and esophageal dysphagia. It is crucial to differentiate between these two forms of dysphagia in affected patients. Patients with oropharyngeal dysphagia have a higher risk of aspiration and aspiration pneumonia, and an accurate initial diagnosis is necessary in such cases.

Patients with oropharyngeal dysphagia may experience difficulty initiating swallowing and report symptoms such as coughing, airway obstruction, nasopharyngeal reflux, aspiration, and the sensation of food remaining in the pharynx. Patients with esophageal dysphagia may find swallowing difficult a few seconds after swallowing initiation and report that the food is stuck in the esophagus. Dysphagia is a relatively common symptom, and its prevalence is increasing.

Esophageal dysphagia is diagnosed by analyzing whether the food causing the swallowing difficulty is solid, liquid, or both; whether the symptoms are progressively worsening or intermittent; the severity of the symptoms; and the presence of accompanying symptoms such as weight loss, chest pain, or reflux. If aspiration is suspected, VFSS or FEES should first be conducted, and if structural abnormalities are suspected, an upper endoscopy should be performed.

Dysphagia is a significant factor affecting prognosis, regardless of the disease type, and can lead to life-threatening complications; therefore, clinicians should approach dysphagia diagnosis and treatment in a systematic manner.

## Figures and Tables

**Table 1:** Common causes of oropharyngeal and esophageal dysphagia.

Oropharyngeal Dysphagia	Esophageal Dysphagia
Neurological	Neuromuscular diseases
Stroke	Achalasia
Parkinson’s disease	Scleroderma
Acquired brain injuries	Connective tissue disease
Neurodegenerative disorders	Mucosal diseases
Brain tumors	Peptic stricture
Neuromuscular	Esophageal rings and webs
Amyotrophic lateral sclerosis	Esophageal tumors
Myasthenia gravis	Radiation therapy
Polymyositis	Infectious esophagitis
Myopathies	Eosinophilic esophagitis
Mechanical	Others
Cervical osteophytes	Foreign bodies
Goiter	Medication adverse effect
Oropharyngeal neoplasms	
Zenker diverticulum	
Others	
Chronic respiratory diseases	
Medication adverse effect	
Radiation therapy	

## Data Availability

No new data were created or analyzed in this study.
